# A Population-Based Cost Analysis of Thoracoscopic Versus Open Lobectomy in Primary Lung Cancer

**DOI:** 10.1245/s10434-016-5125-3

**Published:** 2016-02-04

**Authors:** Bing-Yen Wang, Jing-Yang Huang, Jiunn-Liang Ko, Ching-Hsiung Lin, Yao-Hong Zhou, Chang-Lun Huang, Yung-Po Liaw

**Affiliations:** Division of General Thoracic Surgery, Department of Surgery, Changhua Christian Hospital, Changhua, Taiwan; Chung Shan Medical University, Taichung, Taiwan; School of Medicine, Kaohsiung Medical University, Kaohsiung, Taiwan; Institute of Genomics and Bioinformatics, National Chung Hsing University, Taichung, Taiwan; Department of Public Health and Institute of Public Health, Chung Shan Medical University, Taichung City, 40201 Taiwan; Institute of Medicine, Chung Shan Medical University, Taichung, Taiwan; Division of Chest Medicine, Department of Internal Medicine, Changhua Christian Hospital, Changhua, Taiwan; Department of Respiratory Care, College of Health Sciences, Chang Jung Christian University, Tainan, Taiwan; Department of Cardiothoracic Surgery, Zhejiang University, Mingzhou Hospital, Ningbo, China

## Abstract

**Background:**

Thoracoscopic lobectomy for primary lung cancer has become increasingly popular worldwide due to several advantages over open lobectomy including reduced pain, reduced length of hospital stay, and comparable oncologic outcomes. The costs of thoracoscopic versus conventional open lobectomy have been compared in several studies with variable results. We compared the costs of thoracoscopic versus open lobectomy in lung cancer patients in Taiwan.

**Methods:**

Patients who underwent lobectomy for primary lung cancer from the Taiwan National Health Insurance Research Database (NHIRD) between 2004 and 2010 were identified. Patient characteristics, operative data, and costs for each part of the hospitalization for surgery and 30 days of care after discharge were analyzed.

**Results:**

A total of 5366 patients with complete clinical data who underwent either conventional open lobectomy (*n* = 3166, 59 %) or thoracoscopic lobectomy (*n* = 2200, 41 %) for primary lung cancer were identified from the database. Compared with open lobectomy, thoracoscopic lobectomy was associated with younger age, less comorbidity, shorter anesthesia times, and reduced lengths of hospital stay. Total hospital costs, operative costs, and other costs were significantly higher in the thoracoscopic group. The 30-day after discharge costs were significantly lower in the thoracoscopic group.

**Conclusions:**

Thoracoscopic lobectomy for primary lung cancer in Taiwan was associated with higher total hospital costs but lower 30 days after discharge costs than open lobectomy. These differences may have resulted from higher operative and instrument costs in the thoracoscopic group.

Lung cancer is the leading cause of cancer-related death worldwide.^1^ Treatment for lung cancer is multidisciplinary, and surgery offers the best choice for cure in the early stages of lung cancer. Although some studies have shown comparative prognoses in select patients who underwent sublobar anatomic resection (segmentectomy), lobectomy (either open or thoracoscopic) is still the surgical treatment of choice for resectable lung cancer.^2^,^3^ In addition to acceptable oncologic outcomes, several benefits of thoracoscopic lobectomy over conventional open lobectomy include less acute and chronic pain, shorter duration of hospital stays, fewer complications, and better tolerance of adjuvant chemotherapy.^4^–^6^

Several studies have analyzed the cost of thoracoscopic lobectomy compared with other procedures, such as standard or robot-assisted lobectomy.^7^–^14^ The total cost of thoracoscopic lobectomy was higher than conventional lobectomy in some studies but not in the others.^7^–^10^,^12^–^14^ In this cost-sensitive medical care era, cost may play a role when choosing the type of lobectomy in addition to prognosis and other factors.

The Taiwan National Health Insurance Research Database (NHIRD), managed by the National Health Research Institute of Taiwan, consists of detailed health care data from 99 % of the residents living in Taiwan. We used this population-based database to compare the cost of the thoracoscopic versus open lobectomy approach to lung cancer in Taiwan.

## Patient and Methods

### Data Source

The NHIRD was used as the data source for this study. The released database was used strictly for research purposes, and all information that could potentially identify an individual patient was encrypted. This study was exempt from full review by the Internal Review Board in Changhua Christian Hospital.

The NHIRD, established in 1997, includes nearly 99 % of the 23 million inhabitants of Taiwan and is managed by the National Health Research Institute of Taiwan. It provides clinical health information, including demographic data, primary and secondary diagnoses, clinical data, outpatient and inpatient visits, costs of services and procedures, and treatment patterns. The diagnosis codes used in this study were obtained from the International Classification of Disease, Tenth Revision, Clinical Modification code [ICD-10-CM].

### Patient Selection and Cost Data

Between 2004 and 2010, 65,976 patients were identified with the diagnosis of lung cancer using the diagnostic codes C34.0, C34.1, C34.2, C34.3, C34.8, and C34.9. A total of 13,846 patients were excluded because of incomplete clinical data. Among the remaining 52,130 patients, 5366 (10.3 %) patients underwent either open lobectomy or thoracoscopic lobectomy and were enrolled in the study. Patient characteristics included in this study were age, gender, Charlson score, cell type, and clinical stage. Their histology was described according to the World Health Organization classification. All patients were staged according to the 6th edition of the TNM staging system, published in 1997. The hospitalization and operative data included anesthesia times, lengths of hospital stay, the periods of postoperative chest tube drainage, and surgical mortalities. The cost data included operating room, anesthesia, nursing, pharmacy, intensive care unit, ordinary ward, laboratory, treatments, and other costs. The medical cost for each patient within 30 days after discharge also was extracted from the database. All costs extracted from the database were calculated in New Taiwan dollars (NTD/TWD) and converted to United States dollars (USD) using the conversion, 1 USD = 30 TWD.

### Statistical Analysis

All continuous data were expressed as mean ± standard deviation. The Charlson score was used to quantify preexisting comorbidity as a means of classifying clinical comorbidities, because it is widely used for risk adjustment in administrative datasets.^15^ Surgical mortality was defined as death occurring during the same hospitalization or within 30 days after the operation. Comparisons of categorical data between the two groups were made using *χ*^2^ or the Fisher exact test. Continuous data were compared using the two-tailed *t* test. Statistical analysis was considered significant at *p* < 0.05. The SAS software (SAS System for Windows, version 9.2; SAS Institute, Cary, NC) was used to perform the statistical analysis.

## Results

### Patient Characteristics

Among the 5366 lung cancer patients who underwent lobectomy between 2004 and 2010, 3166 (59 %) underwent conventional open lobectomy and 2200 (41 %) had thoracoscopic lobectomy. Patient demographics and tumor characteristics are listed in Table 1. The average age in the open lobectomy group was 66.63 ± 11.11 years compared with 61.70 ± 11.02 years in thoracoscopic lobectomy group (*p* < 0.0001). There were more males in the open group than in the thoracoscopic (56.95 vs. 45.27 %). The mean Charlson score was higher in the open group compared with the thoracoscopic group (5.58 ± 3.22 vs. 4.87 ± 2.97; *p* < 0.0001). The most common tumor cell type was adenocarcinoma, which accounted for 66.23 % of tumors in the open group and 79.23 % in the thoracoscopic group. The patients in thoracoscopic group had earlier TNM staging compared with patients in open group.

**Table 1:** Patient demographics and tumor characteristics

Characteristics	Open lobectomy	Thoracoscope	*p* value
No. of patients	3166 (59 %)	2200 (41 %)	
Age (year) (mean ± SD)	63.63 ± 11.11	61.70 ± 11.02	<0.0001
Gender			<0.0001
Male	1803 (56.95 %)	996 (45.27 %)	
Female	1363 (43.05 %)	1204 (54.73 %)	
Charlson score	5.58 ± 3.22	4.87 ± 2.97	<0.0001
Cell type			<0.0001
Adenocarcinoma	2097 (66.23 %)	1743 (79.23 %)	
SqCC	670 (21.16 %)	258 (11.73 %)	
Small cell	23 (0.73 %)	10 (0.45 %)	
Large cell	49 (1.55 %)	15 (0.68 %)	
Others	327 (10.33 %)	174 (7.91 %)	
Pathologic stage			<0.0001
T1	1136 (35.88 %)	1187 (53.95 %)	
T2	1611 (50.88 %)	826 (37.55 %)	
T3	268 (8.46 %)	113 (5.14 %)	
T4	126 (3.98 %)	68 (3.09 %)	
Unknown	25 (0.79 %)	6 (0.27 %)	
N			<0.0001
N0	2175 (68.7 %)	1790 (81.36 %)	
N1	333 (10.52 %)	188 (8.55 %)	
N2	560 (17.69 %)	195 (8.86 %)	
N3	69 (2.18 %)	20 (0.91 %)	
Unknown	29 (0.92 %)	7 (0.32 %)	
M			<0.0001
M0	2988 (94.38 %)	2131 (96.86 %)	
M1	163 (5.15 %)	69 (3.14 %)	
Unknown	15 (0.47 %)	0 (0 %)	

*SqCC* squamous cell carcinoma

### Perioperative Details

Perioperative details and patient outcomes are listed in Table 2. The average anesthesia time was longer in the open group compared with the thoracoscopic group (5.55 ± 1.94 vs. 5.40 ± 1.79 h; *p* = 0.033). The average length of hospital stay was 17.49 ± 15.89 days in the open group and 13.00 ± 8.7 days in the thoracoscopic group. The average duration of chest tube placement after operation was longer in the open group compared with the thoracoscopic group (8.68 ± 5.10 vs. 6.44 ± 4.42 days, *p* < 0.001). The surgical mortality was greater in open group compared with the thoracoscopic group (1.04 vs. 0.41 %; *p* = 0.0096).

**Table 2:** Perioperative details and patient outcomes

Variables	Open	Thoracoscope	*p* value
Anesthesia time (hour) (mean ± SD)	5.55 ± 1.94	5.40 ± 1.79	0.0033
Anesthesia time (hour)			0.0377
<4.5	790 (24.95 %)	548 (24.91 %)	
4.5–5	367 (11.59 %)	308 (14 %)	
5–6.5	1159 (36.61 %)	802 (36.45 %)	
≥6.5	850 (26.85 %)	542 (24.64 %)	
Length of stay (days) (mean ± SD)	17.49 ± 15.89	13.00 ± 8.79	<0.0001
Chest tube (days) (mean ± SD)	8.68 ± 5.10	6.44 ± 4.42	<0.0001
Surgical mortality	33 (1.04 %)	9 (0.41 %)	0.0096

### Cost Profile

The detailed hospital costs are listed in Table 3. The total cost of the index hospitalization for lobectomy surgery was higher in the thoracoscopic group ($6,574.1 ± $3,605.9 vs. $6,329.9 ± $4434.3; *p* = 0.026). Regarding breakdown of total cost, only the operative costs and other cost were higher in the thoracoscopic group. The other cost included any instruments or equipment used other than operative costs, such as surgical stapling devices, energy-based vascular sealing and cutting devices, hemostat agents, and tissue adhesives. The 30-day after discharge costs involved any medical costs within 30 days after discharge and included any hospital emergency room visits, outpatient department visits, or any hospital or clinic visits other than the hospital where the patient underwent lobectomy. This cost was significantly higher in the open group compared with the thoracoscopic group ($831.90 ± $1759.90 vs. $612.80 ± $1401.00, *p* < 0.001).

**Table 3:** Cost profile

Variable	Open (USD)	Thoracoscope (USD)	*p* value
Total cost	6329.9 ± 4434.3	6574.1 ± 3605.9	0.0266
Operative	1638.1 ± 310.5	1897.2 ± 362.8	<0.0001
Anesthesia	548.9 ± 211.5	534.1 ± 188.9	0.0071
Nursing	614.7 ± 729.5	447.7 ± 499.4	<0.0001
Pharmacy	501.6 ± 1156.9	349.6 ± 1388.5	<0.0001
ICU	217.2 ± 401.2	144.8 ± 293.2	<0.0001
Ordinary ward	250.4 ± 227.7	192.4 ± 122.7	<0.0001
Labs	893.6 ± 648.2	773.4 ± 551.2	<0.0001
Treatments	496.1 ± 788.3	358.4 ± 538.0	<0.0001
Others	1169.2 ± 1094.8	1876.6 ± 1033.8	<0.0001
30-day after discharge cost	831.9 ± 1759.9	612.8 ± 1401.0	<0.0001

*USD* United States dollars

### Comment

Our study showed that the total hospital costs were higher in the thoracoscopic lobectomy group compared with the open lobectomy group, although the 30-day after discharge costs were significantly lower in the thoracoscopic group. Of the 41 % of patients who underwent thoracoscopic lobectomy for lung cancer treatment in our study, most were females and younger in age compared with the open group.

Lobectomy with radical lymph node dissection is still the mainstream treatment for resectable lung cancers worldwide. In a large study of lung cancer patients in Taiwan, the majority (64.1 %) underwent lobectomy.^16^ The thoracoscopic approach used in lobectomy has become more popular due to greater familiarity with this technique, comparable oncologic outcomes, and distinct advantages over open lobectomy, such as shorter duration of adjuvant chemotherapy, better patient tolerance, less pain, and shorter length of hospital stay.

A study of a general thoracic surgery registry database performed between 1999 and 2006 revealed that only 20 % of patients underwent thoracoscopic approach for primary lung cancer.^17^ Another recent national database analysis found that approximately 39 % of lung cancer patients underwent thoracoscopic lobectomy.^18^ In our study, adenocarcinoma was the predominant cell type in both open and thoracoscopic groups and tumor size tended to be smaller in the thoracoscopic group and the thoracoscopic group also had a lower T stage. The indication of thoracoscopic lobectomy in Taiwan included small peripheral lung cancer and that contributed to smaller tumor size in thoracoscopic group compared with open group.

The cost of thoracoscopic lobectomy compared with conventional open lobectomy has been analyzed in several studies ranging from small sample-sized studies performed at a single institution to large studies using a national database. Some studies have shown lower costs using thoracoscopic lobectomy. Park and colleagues compared robotic, video-assisted thoracoscopic surgery (VATS), and thoracotomy approaches to pulmonary lobectomy and found lower costs and shorter hospital stays in the VATS group.^7^ Casali and Walker reported that thoracoscopic lobectomy had higher surgical costs but lower total costs and this was felt to be related to the shorter hospital stays in the thoracoscopic lobectomy group.^8^ Burfeind and colleagues, in a retrospective analysis of primary lung cancer patients who received either thoracoscopic or open lobectomy, demonstrated less costs for thoracoscopic lobectomy in all phases of patient care.^9^ Swanson et al. also analyzed the cost differences between thoracoscopic and open lobectomy groups from a multi-institutional database and revealed that the VATS approach was less costly with fewer complications, shorter anesthesia times, and shorter hospital stays than open lobectomy.^12^ Fajah and colleagues studied patients who underwent lobectomy not just for lung cancer and further analyzed the 90-day cost after discharge.^13^ They found that the thoracoscopic group had significantly lower total 90-day index hospitalization costs and outpatient costs. In contrast, Gopaldas et al. examined the Nationwide Inpatient Sample database of the United States comparing VATS to open thoracotomy lobectomy and found that the cost of VATS lobectomy tended to be higher than open thoracotomy lobectomy, but the difference was not statistically significant.^10^ The VATS lobectomy group patients had similar hospital stays and more intraoperative complications compared with the open thoracotomy group. Recently, a study reported by Alpay et al. showed that VATS lobectomy costs were greater than costs from thoracotomy lobectomy.^14^ The authors concluded that these findings may have resulted from lower bed fees and higher disposable instrument costs. The total cost of thoracoscopic versus open lobectomy was variable in the above studies. These findings may be explained by different charge policies regarding surgical, anesthesia, and hospital fees, and variable professional and operative fees.

In Taiwan, the medical expenses are regulated by the government under the National Health Insurance Administration, Ministry of Health and Welfare. Although the fees for lobectomy and mediastinal lymph node dissection differ between thoracoscopic versus open approaches, the same procedure in different hospitals costs the same. We found that the operative cost was significant higher in the thoracoscopic group compared with the open group. The most important factor affecting anesthesia cost in our study was anesthesia time. Both anesthesia time and anesthesia cost were significantly higher in the open group compared with the thoracoscopic group. In our study, the length of hospital stay was significantly higher in the open group and that finding could have contributed to the higher cost of ordinary ward, ICU, nursing, and pharmacy costs. The other costs and operative costs were significantly higher in the thoracoscopic group, which made the total cost significantly higher in the thoracoscopic group. Similar to the study performed by Alpay et al., lower bed and manpower costs with higher disposable instrument costs may explain the higher total hospital costs in the thoracoscopic group in our study despite the shorter hospital stays and anesthesia times.^14^

Few studies have analyzed the cost of medical needs after discharge. One of the factors that may have influenced the after-discharge cost in our study was the postoperative complication rate, which may have resulted in more outpatient visits or readmissions. Farjah et al. analyzed the costs up to 90 days after discharge and also found a significantly lower cost in the thoracoscopic group.^13^ As they reported, outpatient use and readmissions accounted for near 16 % of the total 90-day costs after lobectomy. In our study, the 30-day after-discharge cost was significantly lower in the thoracoscopic group. This may be explained by less pain experienced by the patients and, thus, fewer required outpatient visits and analgesic agents along with fewer complications and less need for readmission.

The strength of our study was its large patient size, which included nearly 99 % of the resident population in Taiwan. This factor may have balanced the effects due to the differing economic status among patients and the different hospital volumes (i.e., between a medical center vs. a regional hospital). In addition, the 30-day after-discharge cost included all medical expenses incurred by the same patient at different hospitals.

Our study also had several limitations. The data were conducted on a retrospective cohort, based on diagnostic codes and prescription histories. Registry bias could not be fully excluded, but its influence may have been minimized by the review of the medical expenses conducted by government experts. In addition, although 13,846 (20.9 %) patients were excluded because of incomplete data, compared with other population databases, the percentage of patients with complete data was relatively high in our database.

## Conclusions

This retrospective study from a large Taiwanese database demonstrated that total hospital costs were higher in the thoracoscopic lobectomy group compared with the open lobectomy group and resulted primarily from higher operative and other costs. The 30-day after discharge costs were significantly lower in the thoracoscopic group.
